# Renal Proximal Tubule Cell-specific Megalin Deletion Does Not Affect Atherosclerosis But Induces Tubulointerstitial Nephritis in Mice Fed Western Diet

**DOI:** 10.1101/2024.05.11.592234

**Published:** 2024-06-08

**Authors:** Naofumi Amioka, Michael K. Franklin, Masayoshi Kukida, Hisashi Sawada, Jessica J. Moorleghen, Deborah A. Howatt, Yuriko Katsumata, Adam E. Mullick, Motoko Yanagita, Michelle M. Martinez-Irizarry, Ruben M. Sandoval, Kenneth W. Dunn, Alan Daugherty, Hong S. Lu

**Affiliations:** 1 Saha Cardiovascular Research Center and Saha Aortic Center, University of Kentucky, Lexington, Kentucky, USA; 2 Department of Physiology, University of Kentucky, Lexington, Kentucky, USA; 3 Sanders-Brown Center on Aging, University of Kentucky, Lexington, Kentucky, USA; 4 Department of Biostatistics, University of Kentucky, Lexington, Kentucky, USA; 5 Ionis Pharmaceuticals, Carlsbad, California, USA; 6 Department of Nephrology, Kyoto University Graduate School of Medicine, Kyoto, Japan; 7 Institute for the Advanced Study of Human Biology (WPI-ASHBi), Kyoto University, Kyoto, Japan; 8 Department of Medicine, Indiana University, Indianapolis, Indiana, USA

**Keywords:** megalin, low-density lipoprotein receptor-related protein 2, atherosclerosis, kidney, proximal tubules, angiotensin, mouse

## Abstract

**Background:**

Pharmacological inhibition of megalin (also known as low-density lipoprotein receptor-related protein 2: LRP2) attenuates atherosclerosis in hypercholesterolemic mice. Since megalin is abundant in renal proximal tubule cells (PTCs), the purpose of this study was to determine whether PTC-specific deletion of megalin reduces hypercholesterolemia-induced atherosclerosis in mice.

**Methods:**

Female *Lrp2* f/f mice were bred with male *Ndrg1*-*Cre ERT2* +/0 mice to develop PTC-LRP2 +/+ and −/− littermates. To study atherosclerosis, all mice were to bred to an LDL receptor −/− background and fed a Western diet to induce atherosclerosis.

**Results:**

PTC-specific megalin deletion did not attenuate atherosclerosis in LDL receptor −/− mice in either sex. Serendipitously, we discovered that PTC-specific megalin deletion led to interstitial infiltration of CD68+ cells and tubular atrophy. The pathology was only evident in male PTC-LRP2 −/− mice fed the Western diet, but not in mice fed a normal laboratory diet. Renal pathologies were also observed in male PTC-LRP2 −/− mice in an LDL receptor +/+ background fed the same Western diet, demonstrating that the renal pathologies were dependent on diet and not hypercholesterolemia. By contrast, female PTC-LRP2 −/− mice had no apparent renal pathologies. In vivo multiphoton microscopy demonstrated that PTC-specific megalin deletion dramatically diminished albumin accumulation in PTCs within 10 days of Western diet feeding. RNA sequencing analyses demonstrated the upregulation of inflammation-related pathways in kidney.

**Conclusions:**

PTC-specific megalin deletion does not affect atherosclerosis, but leads to tubulointerstitial nephritis in mice fed Western diet, with severe pathologies in male mice.

## INTRODUCTION

Megalin, also known as low-density lipoprotein receptor-related protein 2 (LRP2), is a 520-kD transmembrane protein that belongs to the low-density lipoprotein (LDL) receptor family. During embryonic development, megalin plays a critical role in brain, cardiovascular, and lung development, as demonstrated by global megalin deficient mice.^1–4^ Megalin becomes most abundant in renal proximal tubule cells (PTCs) after birth,^5^ and it functions primarily as an endocytic receptor in renal PTCs for many ligands including components of the renin-angiotensin system.

The renin-angiotensin system is important for blood pressure regulation and contributes to the pathogenesis of atherosclerosis.^6^ The classic components of the renin-angiotensin system, including AGT, renin, angiotensin-converting enzyme (ACE), angiotensin II (AngII), and AngII type 1 (AT1) receptor, are abundant in kidney.^7, 8^ Our previous study demonstrated that global inhibition of megalin by antisense oligonucleotides (ASO) administration attenuated hypercholesterolemia-induced atherosclerosis in both male and female mice, accompanied by diminished protein abundance of AGT and renin in renal PTCs, as well as AngII production in kidney.^7^ These findings support the hypothesis that megalin contributes to atherosclerosis via its interaction with the renin-angiotensin components in PTCs.

To determine whether region-specific deletion of megalin contributes to atherosclerosis, PTC-LRP2 +/+ and −/− littermates were generated in an LDL receptor −/− background by breeding megalin floxed mice with transgenic mice expressing an inducible *Cre* driven by an N-myc downstream-regulated gene 1 (*Ndrg1*) promoter. In contrast to our findings in both male and female LDL receptor −/− mice administered *Lrp2* ASO,^7^ PTC-specific megalin deficiency did not attenuate hypercholesterolemia-induced atherosclerosis in either sex. Serendipitously, we found that megalin deficiency in PTCs led to tubulointerstitial leukocyte infiltration and tubular atrophy predominantly in male PTC-LRP2 −/− mice with either LDL receptor +/+ or −/− background that were fed a Western diet.

## MATERIALS AND METHODS

### Data availability

Detailed materials and methods are available in this manuscript. Numerical data will be available in the Supplemental Excel File. Bulk RNA sequencing data (raw FASTQ and aligned data) are publicly available at the gene expression omnibus repository (GEO accession number: GSE268879).

### Mice

Female *Lrp2* floxed (*Lrp2* f/f) mice were developed under a contract with the Ingenious Targeting Laboratory using the same strategy reported by Willnow and colleagues.^9^ Female *Lrp2* f/f mice were bred with male *Ndrg1*-*Cre ERT2* +/0 mice.^10^ The breeding strategy for generating *Ndrg1*-*Cre ERT2* 0/0 *Lrp2* f/f (PTC-LRP2 +/+) and *Ndrg1*-*Cre ERT2* +/0 *Lrp2* f/f littermates (PTC-LRP2 −/−) is shown in Supplemental Figure I. To study atherosclerosis, these mice were bred further into LDL receptor −/− background. For mice injected with *Lrp2* ASO, Gen 2.5 ASOs at 6 mg/kg body weight dissolved in sterile PBS were administered via subcutaneous injections once a week for 13 weeks. The injections started 1 week before a Western diet feeding was initiated.

To study renal pathologies, mice were bred to either LDL receptor +/+ or −/− on a C57BL/6J background. DNA was extracted from tails and/or kidneys using Maxwell DNA purification kits (Cat # AS1120; Promega). The information of primers for PCR is shown in Supplemental Table I. *Cre* genotype was determined before weaning and confirmed post-termination. Deletion of *Lrp2* was confirmed in kidney using either PCR, qPCR, or immunostaining of megalin.

All mice were maintained in a barrier facility on a light:dark cycle of 14:10 hours and fed a normal laboratory diet after weaning. To promote *Cre* translocation, mice at 4–6 weeks of age were injected intraperitoneally with tamoxifen (150 mg/kg/day) for 5 consecutive days. Two weeks after the last injection of tamoxifen, mice were fed a diet containing saturated fat (milk fat 21% wt/wt) and cholesterol (0.2% wt/wt; Diet # TD.88137, Inotiv, termed “Western diet”) for 12 weeks to develop atherosclerosis or renal pathologies. In studies investigating the underlying mechanisms of TIN, mice were fed this Western diet for 10 days, 2 weeks, or 12 weeks, depending on the experimental purpose.

Both male and female littermates were used for the experiments reported in this manuscript in accordance with the AHA Statement.^11^ At termination, mice were euthanized using an overdose of a ketamine and xylazine cocktail. All animal experiments in this study were performed according to a protocol approved by the University of Kentucky (Protocol number 2018–2968) or Indiana University (Protocol number 21052 for intravital microscopy) Institutional Animal Care and Use Committee.

### RNA isolation and quantitative PCR (qPCR)

Total RNA was extracted from kidneys using Maxwell^®^ RSC simplyRNA Tissue Kits (Cat # AS1340; Promega) in accordance with the manufacturer’s protocol. Total RNA was transcribed reversely to cDNA using iScript^™^ cDNA Synthesis kits (Cat # 170–8891; Bio-Rad). Quantitative PCR was performed to quantify *Lrp2* mRNA abundance in the kidney using SsoFast^™^ EvaGreen^®^ Supermix kits (Cat # 172–5204; Bio-Rad) on a Bio-Rad CFX96 cycler. Data were analyzed using ΔΔCt method and normalized by the geometric mean of 3 reference genes: *Actb*, *Gapdh*, and *Rplp2*.

### Immunostaining

Immunostaining was performed using Xmatrx^®^ Infinity, an automated staining system (Cat #: AS4000RX; BioGenex), on paraffin-embedded sections to determine the distribution of megalin, CD68, or AGT in the kidney. After fixation using paraformaldehyde (4% wt/vol), kidney samples were incubated in ethanol (70% vo/vol) for 24 hours, embedded into paraffin, and cut at a thickness of 4 µm. Subsequently, sections were deparaffinized using limonene (Cat # 183164; Millipore-Sigma) followed by 2 washes with absolute ethanol (Cat # HC-800–1GAL; Fisher Scientific), and 1 wash with automation buffer (Cat # GTX30931; GeneTex). Deparaffinized sections were incubated with H_2_O_2_ (1% vol/vol; Cat # H325–500; Fisher Scientific) for 10 minutes at room temperature and then antigen retrieval (Cat # HK547-XAK; BioGenex) for 20 minutes at 98 °C. Non-specific binding sites were blocked using goat serum (2.5 % vol/vol; Cat # MP-7451; Vector laboratories) for 20 minutes at room temperature. Sections were then incubated with rabbit anti-megalin antibody (Cat # ab76969; abcam) diluted in primary antibody diluent (Cat #: GTX28208; GeneTex) for 15 min at 40 °C, and rabbit anti-CD68 (E3O7V) antibody (Cat # 97778; Cell Signaling Technology), or AGT (Cat # 28101; IBL-America) overnight at 4 °C. Goat anti-rabbit IgG conjugated with horseradish peroxidase (30 minutes, Cat # MP-7451; Vector Laboratories) was used as the secondary antibody. ImmPACT^®^ NovaRed (Cat # SK4805; Vector) was used as a chromogen, and hematoxylin (Cat # 26043–05; Electron Microscopy Sciences) was used for counterstaining. Slides were coverslipped with mounting medium (Cat # H-5000; Vector). Three negative controls were used for immunostaining: (1) no primary and secondary antibodies, (2) secondary antibody only, and (3) nonimmune rabbit IgG to replace the primary antibody. Images were captured using Axioscan Z1 or 7.

#### Hematoxylin and eosin staining

Paraffin-embedded kidney sections were stained with hematoxylin and eosin. After paraffin removal, sections were stained with eosin (Cat # ab246824, abcam) for 2 minutes and then rinsed with automation buffer. Subsequently, sections were stained with hematoxylin for 30 seconds, rinsed with automation buffer and water, and allowed to air dry. Images were acquired using Zeiss Axioscans (Z1 or 7).

#### Systolic blood pressure measurements

Systolic blood pressure was measured on conscious mice by a non-invasive tail-cuff system (BP-2000, Visitech) following our standard protocol.^12^ Data were collected at the same time each day for three consecutive days before the termination. Criteria for accepted data were systolic blood pressure between 70 and 200 mmHg and standard deviation < 30 mmHg for at least 5 successful recorded data/mouse/day. The mean systolic blood pressure of each mouse from the 3-day measurements was used for data analysis.

#### Quantification of atherosclerosis

Atherosclerotic lesions were traced manually on the intimal surface area of the aorta with an *en face* method in accord with the AHA Statement ^13^ and our standard protocol.^14^ Briefly, the aorta was dissected and fixed in neutrally buffered formalin (10% vol/vol) overnight. The adventitial tissues were removed, and the intimal surface was exposed by longitudinal cuts. Subsequently, the aorta was pinned on a black wax surface, and *en face* images were captured by a digital camera (DS-Ri1; Nikon). Atherosclerotic lesions were traced manually on the images from the ascending aorta to the descending thoracic aorta that was 1 mm distal from the left subclavian artery using a Nikon NIS-Elements software (NIS-Elements AR 5.11.0.). Raw data were verified independently by a senior staff member who was blinded to the identity of the study groups. Atherosclerotic lesion size was presented as percent lesion area showing below:

$$\text{Percent}\;\text{lesion}\;\text{area}=\frac{\text{Atherosclerotic}\;\text{lesion}\left({\text{mm}}^{2}\right)}{\text{Intimal}\;\text{area}\;\text{of}\;\text{the}\;\text{aortic}\;\text{region}\;\left({\text{mm}}^{2}\right)}\times 100$$

### Plasma total cholesterol concentrations

Mouse blood samples were collected with EDTA (1.8 mg/ml) via right ventricle at termination and centrifuged at 400 g for 20 minutes at 4 °C to collect plasma. Plasma total cholesterol concentrations at termination were measured using an enzymatic kit (Cat # 999–02601; FUJIFILM or Cat # C7510–120; Pointe Scientific).

### Urinary Profiling

Urine was collected using metabolic cages (TSE Systems) or LabSand (Braintree Scientific, Inc.). Urine AGT (Cat # ab245718, abcam), RBP4 (Cat # AG-45A-0012YEK-KI01; AdipoGen), KIM-1 (Cat # 213477; abcam), NGAL (Cat # MLCN20; R&D Systems), albumin (Cat # ab207620; abcam), and creatinine (Cat # 1012; Ethos Biosciences) were measured using ELISA kits. Urine renin concentration was measured using a renin activity ELISA kit (Cat # IB59131; IBL-America) after incubating urine samples with additional recombinant mouse AGT.

### In vivo uptake of fluorescent albumin in mouse kidneys

#### Conjugation of fluorescent albumin

Rat serum albumin (Millipore-Sigma, Burlington, MA) was conjugated to Texas Red-X-succinimidyl ester (Thermo Fisher Scientific, Waltham, MA) as described previously.^15^ Briefly, conjugates were prepared per manufacturer's instructions and then dialyzed extensively against 5 x 4 L changes of saline (0.9% wt.vol) over 2 days at 4°C. Purified Texas Red labeled albumin (TR-RSA) was aliquoted into 10 mg tubes, lyophilized, and stored at −80 °C.

#### Mouse preparation/surgery for intravital microscopy

Mice were placed in an induction chamber connected to an anesthesia circuit, dispensing isoflurane at 2% to 4% (vol/vol) at a flow rate of 1 L/min O_2_. Once the mouse was stabilized, the left side of the body above the kidney and the right side of the neck was shaved and cleaned. A 1 cm right ventral incision was made in the neck, the jugular was exposed, and all fat and fascia surrounding were cleared. The anterior end of the jugular was tied using 4–0 silk suture to prevent bleeding. A small nick was made in the jugular vein, and a catheter was slid roughly 1 cm into the jugular vein and secured at the posterior end using 4–0 suture. The catheter was sutured and secured to the skin in three different places. The renal surface was imaged by first making a small incision above the kidney and exteriorizing the kidney gently by gripping the fat from the lower pole and gently pulling out while squeezing the incision behind the kidney. The mouse was transferred over and placed on a second anesthesia circuit delivering isoflurane (2% vol.vol). A two mm^2^ piece of gauze soaked in saline was used to stabilize the kidney in the center of a 50 mm diameter coverslip bottom dish with a 40 mm diameter coverslip (Willco Wells, Electron Microscopy Sciences, Hatfield, PA). Once centered and stabilized, a rectal probe was placed to monitor body temperature which was kept between 36 and 37 °C. A lightweight black plastic cloth was placed over the mouse and a 2.5 cm^2^ space was placed around the mouse, being used to support heating pad set at medium placed over the mouse.

#### Two-photon intravital microscopy

Intravital imaging studies of the renal surface were conducted using a Leica Dive SP-8 (Leica Microsystems, Wetzlar, Germany), with a 40x water immersion objective (NA 1.1). Two-photon excitation at 800 nm was accomplished using a Mai-Tai mode locked laser. Blue, green, and red emissions were collected by the system onto separate 12-bit detectors. Although Texas Red was the only fluorophore utilized in the study, the other channels were collected to acquire a multi-color image of the mouse kidney which includes its autofluorescent signature. To assess accumulation of filtered TR-RSA by proximal tubules, 8 regions containing mostly proximal tubules were marked and background images were collected for each region. A separate region with prominent vessels was selected and ~0.5 mg of TR-RSA was slowly infused while acquiring a time series to assure the fluorescence in the plasma is kept just below saturation. Subsequent images for each region were acquired 10, 30 and 60 minutes after injection of TR-RSA for analysis.

#### Image analyses

TR-RSA accumulation was quantified in images of 8 tubular image fields collected at each time point from the kidneys of PTC-LRP2 +/+ and PTCLRP2 −/− mice. In each field ~9 tubular regions were careful outlined and the mean intensity in the TR-RSA channel was measured using Metamorph image processing software (San Jose, CA). For each time point, TR-RSA fluorescence of each region was quantified as the mean intensity less the background fluorescence of that same region, as measured in the mean intensity measured in the image collected prior to TR-RSA injection. The analyzed data (a total of ~288 tubules from each) were normalized to the highest value (obtained from the PTC-LRP2 +/+ group at 60 minutes)

### Bulk RNA sequencing

RNA was extracted from mouse kidneys using Maxwell^®^ RSC simplyRNA Tissue Kits (Cat # AS1340; Promega) in accordance with the manufacturer’s protocol. Total RNA samples were shipped to Novogene for bulk mRNA sequencing (n=6 biological replicates/group). cDNA library was generated from total mRNA (1 µg) using NEBNext UltraTM RNA Library Prep Kits for Illumina (New England BioLabs). cDNA libraries were sequenced by NovaSeq 6000 (Illumina) in a paired-end fashion to reach more than 1.5M reads. Paired-end read data formatted to FASTQ were mapped to mouse genome mm10 using STAR (v2.5, mismatch=2) and quantified using HTSeq (v0.6.1, -m union).^16, 17^

### Statistical analysis

Data were presented as either mean ± SEM or median with the 25^th^ and 75^th^ percentiles depending on whether the data were analyzed by parametric or non-parametric tests. Normality and homogeneous variance assumptions for raw or log-transformed data with n>5/group were assessed using the Shapiro-Wilk test and the Brown-Forsythe test, respectively. Student’s t-test and one-way analysis of variance (ANOVA) with the Holm-Sidak post-hoc test were used for the data that met both normality and homoscedasticity to compare two-group and multiple-group (n≥3) means. Welch’s t-test was used for data that passed normality test, but failed to satisfy equal variance assumption to compare two-group means. For data did not pass either normality or equal variance test, we applied Mann-Whitney U-test for two-group comparisons or Kruskal-Wallis one-way ANOVA followed by Dunn's method for multiple-group comparisons. Albumin uptake described in Figure 7 was analyzed using a linear mixed effect model with unstructured covariance including genotype, time (10, 30, and 60 minutes), and interaction between genotype and time as main effects and intercept and time as random effects. SigmaPlot version 15 (SYSTAT Software Inc.) was used for statistical analysis except for the data presented in Figures 5 and 7. RNA sequencing data analysis in Figure 5 was performed using the edgeR Bioconductor package (v3.36.0) for differential gene expression (DEG) analysis and the clusterProfiler R Bioconductor package (v4.2.2) for gene ontology (GO) analysis. Data presented in Figure 7 were analyzed using the nlm R package (version 3.1) in R (version 4.2.2). Statistical significancewas set at P<0.05.

## RESULTS

### Validation of inducible PTC-specific megalin deletion in mice

**Figure 1.**
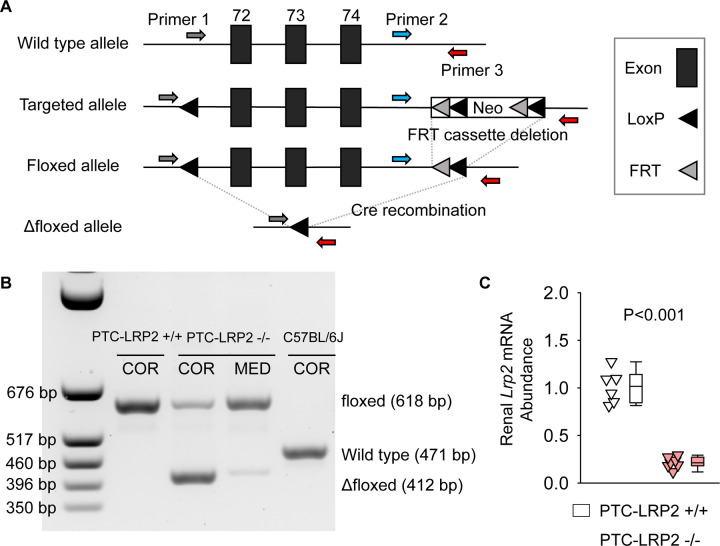

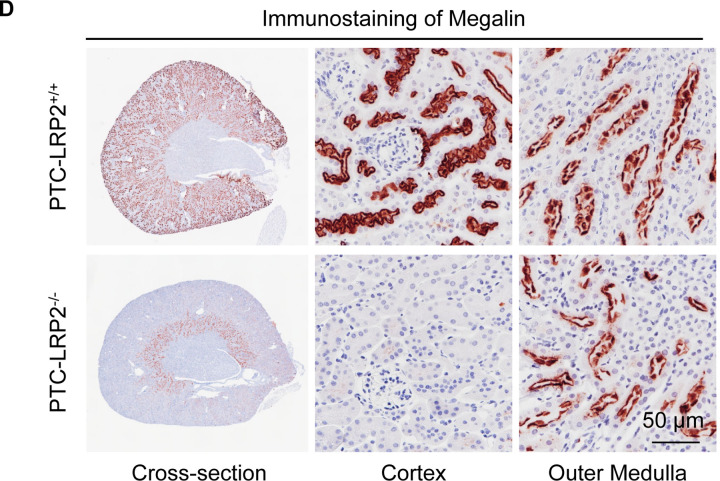
Development and validation of PTC-specific megalin deficient mice. **(A)** Construct map of the *Lrp2* floxed mouse. Three *Lox*P sites were inserted to encompass exons 72–74 of the mouse *Lrp2* gene. One *Lox*P site was inserted in intron 71, and 2 *Lox*P sites were in intron 74. A neo cassette in intron 74 was flanked by the 2 *Lox*P sites and 2 FRT sites in intron 74. After removal of the neo cassette, *Cre* recombination enabled the deletion of exons 72–74 of *Lrp2*, resulting in megalin deletion. **(B)** DNA-PCR using cortex (COR) and medulla (MED) regions of kidneys harvested from male wild-type (WT; C57BL/6J), *Ndrg1*-*Cre ERT2* 0/0 *Lrp2* f/f (PTC-LRP2 +/+), and *Ndrg1*-*Cre ERT2* +/0 *Lrp2* f/f (PTC-LRP2 −/−) mice 2 weeks after the completion of intraperitoneal tamoxifen injection. **(C)** Renal mRNA abundance of *Lrp2* was determined by qPCR (N=6–7/group), and analyzed using Welch’s t-test. **(D)** Immunostaining of megalin illustrated the distribution of megalin in kidneys of PTC-LRP2 +/+ and PTC-LRP2 −/− mice at 2 weeks after the completion of tamoxifen injections.

NDRG1 protein has a predominant abundance in the renal cortex of PTCs (S1 and S2 segments).^10, 18^. PTC-specific megalin deleted mice were generated using *Lrp2* floxed (*Lrp2* f/f) mice and *Cre* transgenic mice expressing a tamoxifen-inducible *Cre* recombinase under the control of *Ndrg1* promoter.^8, 10, 18^ Floxed mice in which exons 72–74 of *Lrp2* were flanked with *LoxP* sites (Figure 1A) were developed using the strategy reported by Leheste and colleagues^9^. Male *Ndrg1*-*Cre ERT2*^+/0^ mice were bred with female *Lrp2* f/f mice to generate F1, F2 and littermates for *in vivo* studies (Figure S1) that had either of the two genotypes: *Ndrg1-Cre ERT2*^0/0^ *Lrp2* f/f (PTC-LRP2 +/+) or *Ndrg1*-Cre *ERT2*^+/0^ *Lrp2* f/f (PTC-LRP2 −/−). Offspring from F2 at 4–6 weeks of age were injected intraperitoneally with tamoxifen (150 mg/kg/day) for 5 consecutive days. Two or 15 weeks after completing the intraperitoneal injection of tamoxifen, cortex and medulla were isolated from kidney tissues to determine floxed allele and deletion of megalin in renal cortex (Figure 1B). qPCR confirmed significant reductions (~80%) of *Lrp2* mRNA in kidney of PTC-LRP2 −/− mice (Figure 1C). As demonstrated by immunostaining for protein distribution, *Cre*-*LoxP* recombination led to deletion of megalin in S1 and S2 of PTCs, but its presence in S3 remained (Figure 1D).

Our and others’ previous studies demonstrated that AGT, the substrate of all angiotensin peptides, in S1 and S2 of PTCs is derived primarily from hepatocytes, whereas AGT protein in S3 of PTCs is derived from kidney.^7, 19^ In the absence of megalin in S1 and S2 of PTCs, AGT became abundant in S3 of PTCs, while its presence in S1 and S2 of PTCs was minimal (Figure S2).

### PTC-specific megalin deletion did not affect atherosclerosis in hypercholesterolemic mice

**Figure 2.**
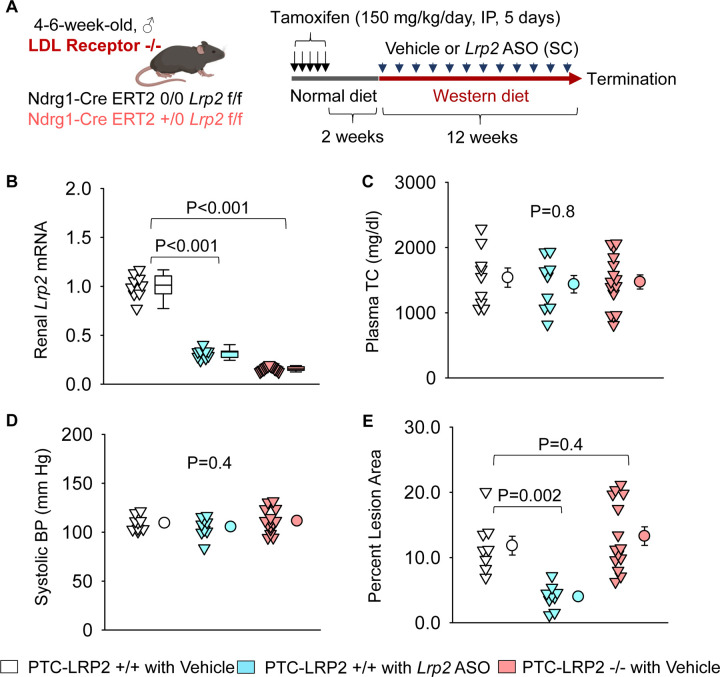
PTC-specific deletion of megalin did not attenuate hypercholesterolemia-induced atherosclerosis. **(A)** Experimental protocol: Four to 6-week-old male mice in an LDL receptor −/− background received intraperitoneal (IP) injections of tamoxifen for 5 consecutive days. Two weeks after completing the tamoxifen injection, all study mice were fed a Western diet for 12 weeks. The study mice received subcutaneous (SC) injections of PBS (Vehicle) or *Lrp2* antisense oligonucleotides (*Lrp2* ASO, 6 mg/kg/week) started 1 week prior to Western diet feeding. **(B)** Renal *Lrp2* mRNA abundance was determined using qPCR. **(C)** Plasma total cholesterol (TC) concentrations were measured using an enzymatic method. **(D)** Systolic blood pressures(BP) were measured using a tail-cuff system. **(E)** Percent atherosclerotic lesion area was quantified using an *en face* approach. Statistical analysis: Kruskal-Wallis one-way ANOVA on ranks followed by the Dunn method **(B)** and one-way ANOVA followed by the Holm-Sidak method **(C-E)**.

Following validation of the phenotype of genetically manipulated mice, we determined the effects of PTC-specific megalin deletion on blood pressure and atherosclerosis (Figure 2, Figure S3). PTC-LRP2 +/+ and PTC-LRP2 −/− littermates in an LDL receptor −/− background were injected with tamoxifen at 4–6 weeks of age. Two weeks after the completion of tamoxifen injections, Western diet feeding was started and continued for 12 weeks. Our previous study reported that *Lrp2* ASO reduced hypercholesterolemia-induced atherosclerosis in LDL receptor −/− mice.^7^ Therefore, subcutaneous injection of *Lrp2* ASO (6 mg/kg/week) to one group of PTC-LRP2 +/+ littermates was used as a positive control for this atherosclerosis study. Since our previous study has confirmed that control ASO showed comparable results as PBS (the solvent for ASOs) on blood pressure and atherosclerosis, PBS injection was used as the negative control (vehicle) of *Lrp2* ASO. PTC-LRP2 +/+ mice injected with *Lrp2* ASO and PTC-LRP2 −/− mice injected with vehicle showed significant reductions of renal *Lrp2* mRNA abundance, compared to PTC-LRP2 +/+ mice in both sexes (Figure 2B, Figure S3A). In contrast to the significant decrease of *Lrp2* mRNA abundance, there was only a modest reduction in megalin protein observed in PTC-LRP2 +/+ mice injected with *Lrp2* ASO, compared to the absence of megalin protein in S1 and S2 of PTC-LRP2 −/− littermates (Figure S4).

Plasma total cholesterol concentrations were not different among the three groups (Figure 2C, Figure S3B). Also, inhibition by *Lrp2* ASO or PTC-specific deletion of megalin did not change systemic blood pressure in either sex (Figure 2D, Figure S3C). Consistent with our previous findings,^7^ inhibition of megalin globally by *Lrp2* ASO significantly suppressed atherosclerosis development in both male and female PTC-LRP2 +/+ mice (Figure 2E, Figure S3D). However, atherosclerotic lesion size was not different between PTC-LRP2 +/+ and PTC-LRP2 −/− littermates injected with PBS.

### PTC-specific megalin deficiency led to tubulointerstitial nephritis in male LDL receptor −/− mice fed Western diet

**Figure 3.**
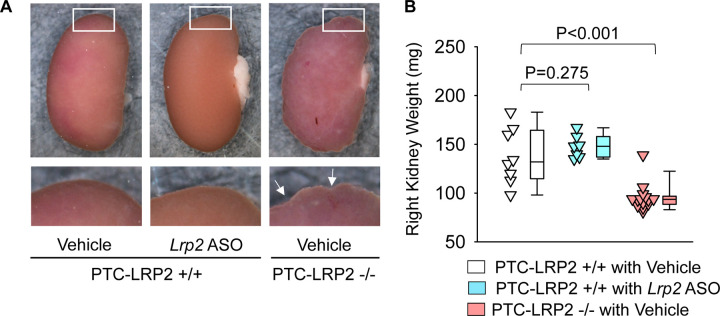

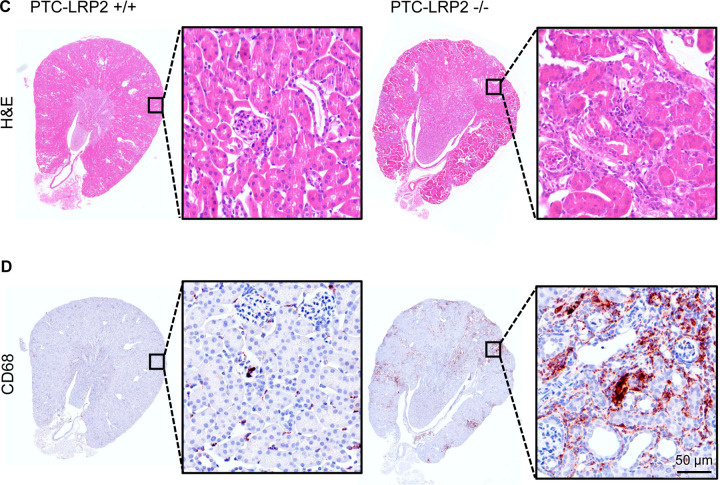
PTC-specific megalin deletion led to TIN in male LDL receptor −/− mice fed a Western diet. Four to 6-week-old male mice on an LDL receptor −/− background received intraperitoneal injections of tamoxifen for 5 consecutive days. Two weeks after completing the tamoxifen injection, all study mice were fed a Western diet for 12 weeks. The study mice received subcutaneous (SC) injections of PBS (Vehicle) or *Lrp2* antisense oligonucleotides (*Lrp2* ASO, 6 mg/kg/week) started 1 week prior to Western diet feeding. **(A)** Gross appearance of kidneys and **(B)** right kidney weight at termination. Statistical analyses: one-way ANOVA followed by the Holm-Sidak test after log-transformation. **(C)** Hematoxylin and eosin (H&E) staining, and **(D)** immunostaining of CD68 in kidney (N=7–14 per group).

Surprisingly, during necropsy we noted that all male littermates with PTC-specific megalin deficiency exhibited smaller kidney weight and abnormal morphology with distinctly irregular surface (Figure 3A and 3B). We did not observe overt morphological changes in male wild type littermates injected with *Lrp2* ASO. Histological analysis was performed to illustrate the features of the renal pathologies in male PTC-LRP2 −/− mice fed a Western diet, compared to their PTC-LRP2 +/+ littermates. With hematoxylin and eosin staining (Figure 3C), the cortex and the outer medulla parts of kidneys obtained from male PTC-LRP2 +/+ mice were uniform, whereas loss of eosin staining presented in a radial pattern in the cortex of kidneys from male PTC-LRP2 −/− mice. This pattern was associated predominantly with PTC atrophy (Figure 3C). Additionally, there are many cells accumulated in the interstitial areas. Immunostaining of CD68 revealed macrophage accumulation (Figure 3D). Overall, these pathological features are consistent with tubulointerstitial nephritis, a common cause of acute kidney injury that can progress to chronic kidney disease.^20^

In contrast to the severe pathological changes observed in male PTC-LRP2 −/− mice fed the Western diet, kidneys harvested from female PTC-LRP2 −/− mice after 12 weeks of the same diet (Figure S5) did not exhibit morphological alterations. There were no discernable differences in kidney weight between the two genotypes. H&E staining did not detect obvious proximal tubule atrophy in female PTC-LRP2 −/− mice. Immunostaining revealed sparsely accumulated CD68+ cells in the interstitial space in both PTC-LRP2 +/+ and PTC-LRP2 −/− mice (Figure S5).

To determine whether the renal pathologies were related to Western diet feeding, male mice with PTC-specific deletion of megalin were fed a normal laboratory diet for 15 weeks after completing injections of tamoxifen (Figure S6). Plasma total cholesterol concentrations and kidney weight were not different between PTC-LRP2 +/+ and −/− mice. Although deletion of megalin in S1 and S2 of PTCs was evident in male PTC-LRP2 −/− mice, no apparent renal pathologies were observed (Figure S6).

**Figure 4.**
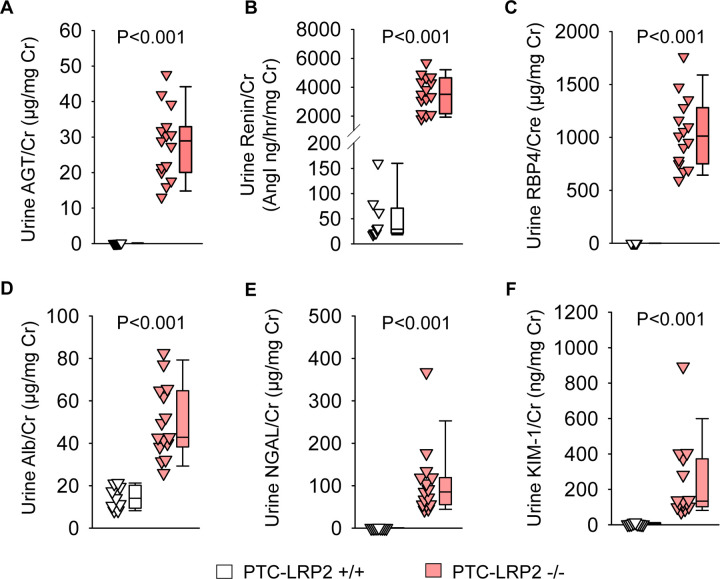
PTC-specific megalin deletion increased renal PTC injury markers in male LDL receptor −/− mice fed a Western diet. Four to 6-week-old male mice in an LDL receptor −/− background received intraperitoneal injections of tamoxifen for 5 consecutive days. Two weeks after completing the tamoxifen injection, all study mice were fed a Western diet for 12 weeks. Urine was collected before termination. AGT **(A)**, renin **(B)**, RBP4 **(C)**, albumin **(D)**, NGAL **(E)**, and KIM-1 **(F)** in urine were measured using ELISA kits and normalized by urine creatinine concentrations. Statistical analysis: Mann-Whitney U-test **(A, C-F)** and Welch’s t-test **(B)** because data presented in **(B)** passed the normality but did not pass equal variance test.

In the absence of megalin in PTCs, AGT and renin were present in high concentrations in urine (Figure 4A and B, Figure S7A and B). Retinol-binding protein 4 (RBP4), a functional biomarker of PTCs, is regulated by megalin.^7, 21^ RBP4 in urine was not detectable in PTC-LRP2 +/+ mice; however, it was present in high concentrations in PTC-LRP2 −/− mice, irrespective of sex (Figure 4C, Figure S7C). Albumin, filtered through glomeruli, is normally taken up by renal PTCs in a megalin and cubilin-mediated manner.^22^ The ratio of urinary albumin to urine creatinine was increased by PTC-specific megalin deficiency in both male and female mice (Figure 4D, Figure S7D). Urine neutrophil gelatinase-associated lipocalin (NGAL) and kidney injury molecule-1 (KIM-1) are biomarkers representing impaired proximal tubules.^23^ Pronounced increases of urinary NGAL and KIM-1 were observed in both sexes of PTC-LRP2 −/− mice (Figure 4E and F, Figure S7E and F). Overall, there were increased concentrations of many megalin ligands in the urine of PTC-LRP2 −/− mice.

### PTC-specific megalin deficiency augmented inflammation-related transcriptomes in kidneys of male LDL receptor −/− mice fed Western diet

**Figure 5.**
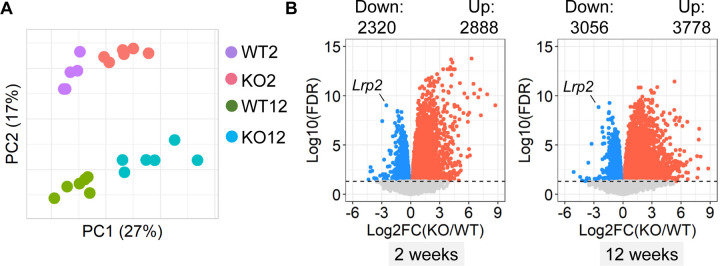

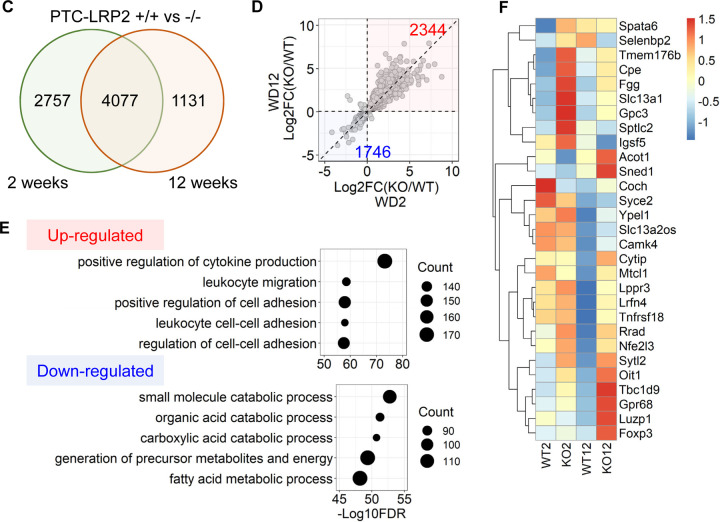
PTC-specific megalin deficiency augmented inflammation-related transcriptomes in male mice fed a Western diet. Five-week-old male mice in an LDL receptor −/− background received intraperitoneal (IP) injections of tamoxifen for 5 consecutive days. Two weeks after completing the tamoxifen injection, all study mice were fed a Western diet for either 2 or 12 weeks. Renal cortex from each mouse was collected to isolate RNA and bulk RNA sequencing was performed subsequently. WT = PTC-LRP2 +/+ mice and KO = PTC-LRP2 −/− mice. **(A)** Principal component analysis (PCA) of transcriptomes. WT2 = PTC-LRP2 +/+ mice fed a Western diet for 2 weeks; KO2 = PTC-LRP2 −/− mice fed a Western diet for 2 weeks; WT12 = PTC-LRP2 +/+ mice fed a Western diet for 12 weeks; and KO12 = PTC-LRP2 −/− mice fed a Western diet for 12 weeks; **(B)** Volcano plot depicting differentially expressed genes (DEGs) between the two genotypes at 2 or 12 weeks of Western diet feeding.Five-week-old male mice in an LDL receptor −/− background received intraperitoneal (IP) injections of tamoxifen for 5 consecutive days. Two weeks after completing the tamoxifen injection, all study mice were fed a Western diet for either 2 or 12 weeks. Renal cortex from each mouse was collected to isolate RNA and bulk RNA sequencing was performed subsequently. WT = PTC-LRP2 +/+ mice and KO = PTC-LRP2 −/− mice. **(C)** Overlap and **(D)** correlation of DEGs between the two genotypes after either 2 or 12 weeks of a Western diet feeding. **(E)** Gene ontology enrichment analysis using the overlapping DEGs. **(F)** Heatmap with Zscored coloring displaying genes associated with inflammation identified through deviance analysis. N=6/group.

To explore potential mechanisms by which deletion of PTC-specific megalin deletion induced renal pathologies, 2 weeks after tamoxifen induction, male PTC-LRP2 +/+ and PTC-LRP2 −/− littermates were fed a Western diet for either 2 weeks (representing early pathological status) or 12 weeks (representing advanced pathological status). Gross morphology was not apparently different between the two genotypes when the Western diet was fed only for 2 weeks. Renal cortex from each mouse was collected to isolate RNA and bulk RNA sequencing was performed subsequently. Transcriptomic patterns of the two genotypes between the two time points (2 versus 12 weeks of Western diet feeding) were different, as illustrated by principal component analysis (Figure 5A). PTC-specific megalin deletion resulted in up- and downregulation of over 2000 genes (Figure 5B), respectively, with a total of 4,077 genes exhibiting significant changes in their expression when fed Western diet for either 2 or 12 weeks (Figure 5C). Among all changed genes, PTC-specific megalin deletion led to 2,344 genes being upregulated and 1,746 genes being downregulated (Figure 5D). Enrichment analysis using the common DEGs demonstrated that inflammation-related pathways were upregulated, whereas metabolism-related pathways were downregulated in response to PTC-specific megalin deficiency in mice fed Western diet (Figure 5E and F). Based on the findings from both pathological and transcriptomic assessments at an early stage (2 weeks of Western diet feeding) and a chronic stage (12 weeks of Western diet feeding) of the renal phenotypes, inflammation occurred shortly after starting Western diet feeding. This was also consistent with macrophage accumulation observed in the interstitial space of kidneys from PTC-LRP2 −/− mice fed Western diet.

### PTC-specific megalin deficiency led to tubulointerstitial nephritis in male C57BL/6J mice fed Western diet

**Figure 6.**
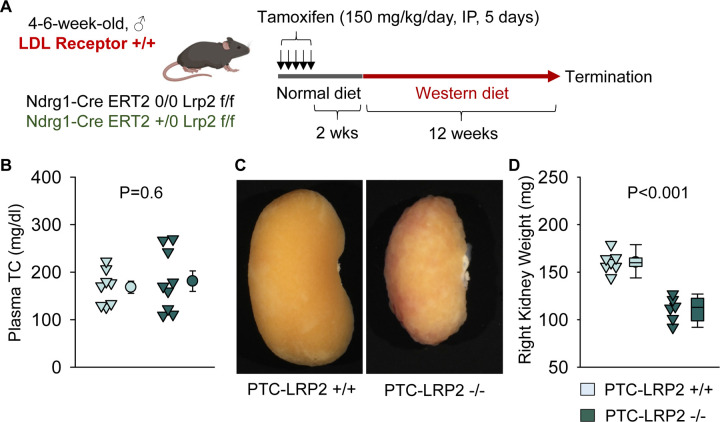

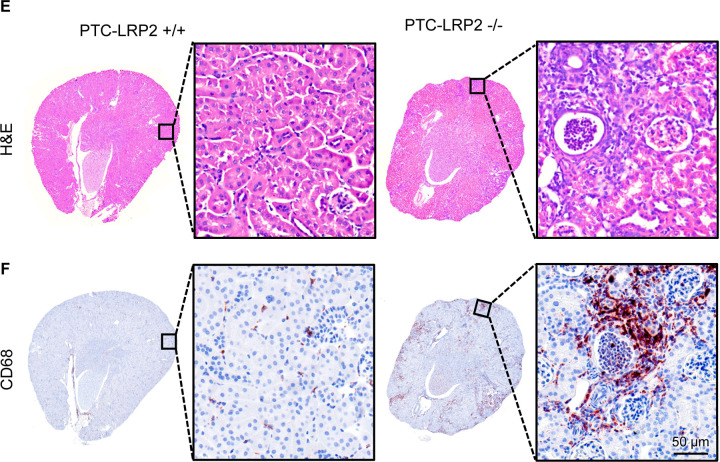
PTC-specific megalin deletion led to TIN in male LDL receptor +/+ mice fed a Western diet. **(A)** Experimental protocol: Five-week-old male LDL receptor +/+ mice on a C57BL/6J background received intraperitoneal (IP) injections of tamoxifen for 5 consecutive days. Two weeks after completing the tamoxifen injection, all mice were fed a Western diet for 12 weeks. **(B)** Plasma total cholesterol (TC) concentrations were measured using an enzymatic method. **(C)** Gross appearance of kidney, **(D)** weight of right kidney, **(E)** hematoxylin and eosin (H&E), and **(F)** immunostaining of CD68 in kidneys after termination. N=6–9/group. Statistical analysis: Studenťs t-test **(B)** or Mann-Whitney Rank-Sum test **(D)**.

Striking renal pathologies were observed in male PTC-LRP2 −/− mice with an LDL receptor −/− background fed the Western diet, but not in male mice fed a normal laboratory diet for comparable intervals. To determine whether the renal pathologies were dependent on LDL receptor deficiency, we repeated the study in LDL receptor +/+ mice that were on a strain- equivalent background of C57BL/6J (Figure 6A). PTC-LRP2 +/+ and PTC-LRP2 −/− littermates in LDL receptor +/+ background were injected with tamoxifen at 4–6 weeks of age. Two weeks after completion of tamoxifen injections, Western diet feeding was started and continued for 12 weeks. PTC-specific megalin deficiency in male LDL receptor +/+ mice showed abnormal renal morphology, proximal tubule atrophy, and interstitial inflammation (Figure 6B–F) that were consistent with the pathology observed in male PTC-LRP2 −/− mice in an LDL receptor −/− background, although these mice were not hypercholesterolemic. These data support the notion that Western diet feeding, rather than hypercholesterolemia, induces renal pathologies in male PTC-LRP2 −/− mice.

The renal pathologies, as determined by kidney weight, H&E staining, and immunostaining of CD68, were not apparent in female PTC-LRP2 −/− mice with the LDL receptor +/+ background fed Western diet (Figure S8).

### PTC-specific megalin deficiency led to impaired accumulation of fluorescently labeled albumin in male C57BL/6J mice fed a Western diet

**Figure 7.**
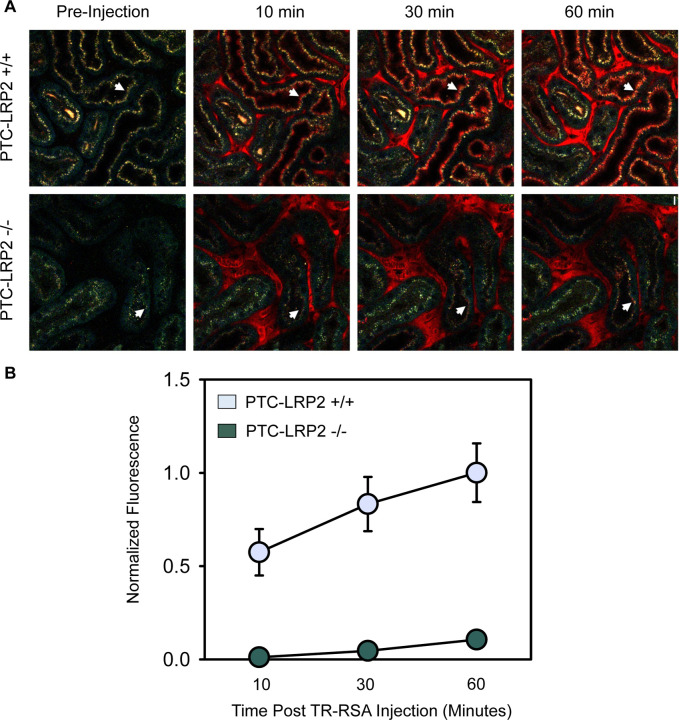
PTC-specific megalin deficiency led to impaired uptake of fluorescent-albumin in male mice fed a Western diet. Four to 6-week-old male mice on an LDL receptor +/+ background received intraperitoneal injections of tamoxifen for 5 consecutive days. Two weeks after completing the tamoxifen injection, all study mice were fed a Western diet for 10 days. Multiphoton intravital microscopy was conducted to quantify PTC accumulation of albumin. **(A)** In vivo multiphoton fluorescence images were collected from the kidneys of PTC-LRP2 +/+ and PTC-LRP2 −/− mice prior to intravenous injection of Texas Red-labeled rat serum albumin (TR-RSA) (Top row), and 10, 30 and 60 minutes after injection. Arrows indicate examples of the same PTC regions imaged over time. **(B)** Quantitative analysis of albumin PTC uptake in images collected from PTC-LRP2 +/+ and PTCLRP2 −/− mice. Statistical analysis: a linear mixed effects model with unstructured covariance matrix. P=0.0034 between the two genotypes at 10 minutes, and P<0.001 for interaction between genotype and time.

Disruption of megalin function in PTC-LRP2 −/− mice in LDL receptor +/+ background was verified by quantitative intravital microscopy of proximal tubule uptake of albumin, a known ligand of megalin. After 10 days of Western diet feeding, male PTC-LRP2 +/+ and PTC-LRP2 −/− littermates had three-dimensional image volumes collected in vivo, before and at 10, 30, and 60 minutes, respectively, after intravenous injection of Texas Red-labeled rat serum albumin (TR-RSA). Representative fields collected from PTC-LRP2 +/+ and PTC-LPR2 −/− mice at each interval are shown in Figure 7A. Quantitative analyses demonstrated that the rate of initial uptake (measured at 10 minutes) was reduced ~47-fold in PTC-LPR2 −/− mice (Figure 7B). Interestingly, significantly less punctate lysosomal autofluorescence (as shown by the pre-injection images in Figure 7A) was noted in PTC-LRP2 −/− mice, suggesting that loss of megalin might have decreased uptake of other endogenous ligands. In addition to the rapid disruption of albumin accumulation observed by intravital microscopy, 2 weeks of Western diet feeding also led to remarkable inflammation, as demonstrated by the accumulation of CD68 positive cells interstitially (Figure S9).

## DISCUSSION

The primary objective of this study was to investigate the role of megalin in PTCs in hypercholesterolemia-induced atherosclerosis. Contrary to our initial hypothesis, deletion of megalin in S1 and S2 of PTCs did not reduce atherosclerosis in LDL receptor −/− mice. However, serendipitously, we observed that deletion of megalin in these two segments of PTCs led to TIN. There are several significant and novel findings shown in this study: (1) PTC-specific deletion of megalin resulted in TIN in male mice fed a Western diet, but not in mice fed a normal laboratory diet, (2) Western diet-induced TIN in PTC-LRP2 −/− mice was independent of hypercholesterolemia, (3) PTC-specific megalin deletion-induced TIN was severe in male mice, but not evident in female mice, and (4) PTC-specific megalin deletion resulted in rapid onset of interstitial inflammation following the initiation of Western diet feeding. This inflammation was accompanied by pronounced functional impairment of PTCs to uptake albumin, a prominent ligand of megalin.

In adult mice, megalin is mainly present in renal PTCs, while other tissues and organs either lack or have very low abundance of megalin.^7^ Megalin is necessary to retain AGT and renin in renal PTCs, where high concentrations of AngII, a major contributor to blood pressure regulation and atherosclerosis, are present.^7, 24–26^ Our previous and the present studies demonstrated consistently that ASO-induced deletion of megalin reduced atherosclerosis.^7^ Therefore, it was initially anticipated that PTC-derived megalin would be the primary source contributing to atherosclerosis. However, the present study involving a large number of animals, including both males and females, do not support this initial hypothesis. Effects of tamoxifen on atherosclerosis have been implicated in previous reports.^27^ Since all study mice were administered an equivalent amount of tamoxifen, the present study does not support the notion that transient tamoxifen administration affected atherosclerosis. Although the basis of PTC-specific deletion of megalin not changing atherosclerosis development is unclear, there are multiple differences between *Lrp2* ASO administration and PTC-specific megalin deficiency that may contribute to the conflicting findings. While both *Lrp2* ASO administration and PTC-specific megalin deficiency led to similar reductions of *Lrp2* mRNA abundance, the protein abundance reduction of megalin differed between the ASO pharmacological approach and the genetic deletion approach. Specifically, although megalin protein abundance from S1 and S2 to S3 was reduced, it was still detectable in mice injected with *Lrp2* ASO, whereas megalin protein in S1 and S2 was abolished in genetically engineered PTC-LRP2 −/− mice. In addition, megalin was abundant in S3 following its genetic deletion in S1 and S2 of PTCs. It is unclear whether ablation of megalin in S1 and S2 is detrimental, or the high abundance of megalin in S3 plays a critical role in contributing to atherosclerosis. Pax8 promoter in the *Pax8-etTA* transgenic mouse line targets all renal tubule cells including proximal and distal tubules as well as collecting duct system.^28^ Unfortunately, there are no *Cre* promoters that target S3 specifically. Second, although loss of AGT, renin, RBP4, and albumin were also detected in mice injected with *Lrp2* ASO, no severe renal dysfunction was observed in these mice. In contrast, genetic deletion of megalin in S1 and S2 of PTCs in male mice led to severe renal impairment when fed Western diet. Since persistent kidney damage is an independent risk factor for atherosclerosis,^29–31^ it is possible that severe renal dysfunction could contribute to atherosclerosis development in PTC-LRP2 −/− mice.

The most surprising finding in this study is that PTC-specific megalin deletion resulted in TIN in male mice fed a Western diet, but not when fed a normal laboratory diet. This striking phenotype was observed in both LDL receptor +/+ and LDL receptor −/− mice that are on a C57BL/6J background. Plasma total cholesterol concentrations in LDL receptor −/− mice fed a Western diet were greater than 1,000 mg/dL, but were less than 200 mg/dL in LDL receptor +/+ mice fed a Western diet. These results support the concept that an currently unidentified constituents of Western diet are contributing to PTC-specific megalin deficiency-induced TIN, whereas hypercholesterolemia *per se* is not essential. The RNA sequencing analyses conducted on kidney samples from mice fed a Western diet for 2 or 12 weeks revealed notable differences between male PTC-LRP2 +/+ and PTC-LRP2 −/− mice, particularly evident in decreased gene expressions related to catabolism and metabolism and increased gene expression associated with inflammation. Reduced catabolic and metabolic gene expression may be aligned with the loss of megalin ligands in urine of PTC-LRP2 −/− mice or loss of physiological function to maintain catabolic and metabolic homeostasis. Increased inflammatory gene expression was associated with pronounced accumulation of immunostained macrophages in the renal interstitial regions of PTC-LRP2 −/− mice. This finding starkly contrasts with the sparse presence of macrophages in the kidney of PTC-LRP2 +/+ mice, as well as in mice of either genotype fed a normal laboratory diet. Inflammation is recognized as a hallmark of TIN.^20^ In the present study, the early onset of inflammation, as evidenced after just 2 weeks of Western diet feeding in PTC-LRP2 −/− mice, indicates that inflammation may be a potential causal factor contributing to the renal pathologies observed in these mice. However, the precise mechanisms triggering TIN remain unclear.

The findings of renal pathologies in PTC-LRP2 −/− mice fed Western diet conflict with those reported by Kuwahara and colleagues that kidney-specific reductions of megalin improved high-fat diet-induced renal pathological impairment.^32^ In that study, *Lrp2* floxed mice were bred with transgenic mice expressing *Cre* under the control of a human *Apoe* promoter which led to megalin deletion in a mosaic pattern in mouse renal cortex, with ~50–60% of megalin remaining in most PTCs.^9, 19, 24, 32^ C57BL/6J mice fed a high-fat diet (60% calories/wt from fat) for 12 week resulted in modest hypertrophy, lipid peroxidation, and cellular senescence of PTCs. These renal changes were attenuated with the mosaic deletion of megalin. In addition to the different diet contents, one major difference between our study and the previously reported studies is the magnitude of megalin deletion in PTCs. *Cre* transgene driven by a *Ndrg1* promoter led to ablation of megalin in S1 and S2 of PTCs, but *Apoe*-*Cre* transgene led to only ~40–50% deletion of megalin in most PTCs. Megalin mediates endocytosis of a variety of molecules in PTCs such as RBP4 and albumin; however, it may also mediate uptake of harmful components in the saturated fat-enriched diet that should be eliminated to maintain the physiological homeostasis. The renal pathologies observed in PTC-LRP2 −/− mice fed the Western diet were not detected in C57BL/6J mice administered *Lrp2* ASO, which inhibits megalin globally, but did not abolish megalin in S1 and S2 of PTCs. Megalin is abundant in S3 of PTCs in PTC-LRP2 −/− mice. Whether the absence of megalin in S1 and S2 is detrimental or the highly abundant megalin in S3 is crucial in promoting TIN remains unclear. Development of mice expressing *Cre* specifically in S3 would provide valuable insights into resolving this dilemma in the future.

In addition to the renal pathologies observed in this study, adenine diet-induced TIN in mice or rats has been used frequently to study mechanisms and potential therapeutic strategies of TIN.^33, 34^ In this model, adenine in the diet leads to obstruction of the urinary tract due to the precipitation of adenine crystals. Consequently, the obstructed urinary tract results in injury to renal tubules including proximal tubules.^33, 35^. This model presents several limitations, including the significant variability in the severity of pathologies, which challenges its consistency in studying TIN. In contrast, the renal pathologies observed in the present study was remarkably consistent, occurring in all male mice with PTC-specific megalin deletion fed a Western diet – a diet that represents the current diet habits in many Western countries. Notably, the striking renal pathologies observed in PTC-LRP2 −/− mice mirrored kidney pathologies found in various cardiovascular diseases, such as hypertension and diabetes.^36^ However, no preclinical and clinical studies have reported whether megalin impairment in PTCs manifests under these prevalent cardiovascular disease conditions. Additionally, PTC-specific megalin deletion in mice represents some relatively rare immune complex conditions found in humans. Recent human case reports or observational studies have identified manifestations of TIN related to autoantibodies against megalin on the PTC brush borders, a condition termed “anti-megalin nephropathy”.^37–42^. This nephropathy presents as severe tubulointerstitial injury and inflammation, with no or mild glomerular impairment, yet ultimately progresses to advanced renal disease. Although the potential connection between fat-enriched diet feeding in PTC-LRP2 −/− mice and this human condition is unclear, megalin impairment-induced TIN in mice has potential relevance to humans, highlighting its importance in exploring potential molecular mechanisms underlying human diseases.

Although urinary protein concentrations were similarly severe between male and female mice, the pathological changes were not evident in female PTC-LRP2 −/− mice. This sex difference is also noted in the mouse model with adenine-induced TIN.^43^ It is well known that cardiovascular diseases have strong sex differences.^11^ Some human studies also suggest that renal dysfunction progresses more slowly in women than in men, although conflict findings exist.^44–48^. Sex hormones, such as estrogen, are potential contributors to this sex dimorphism,^49, 50^ but their impact on the development and pathogenesis of TIN has not been defined. Future studies will be needed to explore the potential mechanisms by which PTC-specific megalin deletion in male mice leads to more severe TIN.

In summary, deletion of megalin specifically in S1 and S2 of PTCs failed to mitigate hypercholesterolemia-induced atherosclerosis, but instead induced TIN with severe pathological changes in male mice. The consumption of a Western diet exerted a crucial role in triggerring the observed TIN. Future studies aim to understand the potential molecular mechanisms and pathogenesis of TIN associated with megalin deletion in S1 and S2 of PTCs, related sexual dimorphism, as well as its long-term impact on kidney and cardiovascular functions.

## Supplementary Material

Supplement 1

## Highlights

1. Deletion of megalin specifically in S1 and S2 of PTCs (proximal tubules) does not reduce atherosclerosis in hypercholesterolemic mice, irrespective of sex.
2. Deletion of megalin in S1 and S2 of PTCs induces TIN (tubulointerstitial nephritis) with severe renal pathological changes in male mice.
3. PTC-specific megalin deficiency-induced TIN occurs in both male LDL receptor −/− and LDL receptor +/+ mice fed a Wetsern diet.
4. The consumption of a Western diet exerts a crucial role in triggerring the observed TIN in male mice with PTC-specific megalin deletion.

## Abbreviations

AngII: angiotensin II
ASO: antisense oligonucleotides
LDL: low-density lipoprotein
LRP2: low-density lipoprotein receptor-related protein 2
PTC: proximal tubule cell
