# Supplementary material for: Renal Proximal Tubule Cell-specific Megalin Deletion Does Not Affect Atherosclerosis But Induces Tubulointerstitial Nephritis in Mice Fed Western Diet
Source: bioRxiv. 2024 Jun 8:2024.05.11.592234. Originally published 2024 May 14. Preprint. [Version 2] doi: 10.1101/2024.05.11.592234 (PMC11118422; doi:10.1101/2024.05.11.592234)
Supplement: Supplementary file 1 [file media-1.pdf]

SUPPLEMENTAL MATERIALS

Renal Proximal Tubule Cell-specific Megalin Deletion Does Not Affect Atherosclerosis But Induces Tubulointerstitial Nephritis in Mice Fed Western Diet

Naofumi Amioka,[1,*] Michael K. Franklin,[1,*] Masayoshi Kukida,[1,*] Hisashi Sawada[1,2], Jessica J. Moorleghen[1], Deborah A. Howatt,[1] Yuriko Katsumata[3,4], Adam E. Mullick,[5] Motoko Yanagita,[6,7] Michelle M. Martinez-Irizarry,[8] Ruben M. Sandoval Jr,[8] Kenneth W. Dunn,[8] Alan Daugherty,[1,2] Hong S. Lu[1,2]

[1] Saha Cardiovascular Research Center and Saha Aortic Center, [2] Department of Physiology, [3] Sanders-Brown Center on Aging, [4] Department of Biostatistics, University of Kentucky, Lexington, Kentucky, USA
[5] Ionis Pharmaceuticals, Carlsbad, California, USA

[6] Department of Nephrology, Kyoto University Graduate School of Medicine, [7] Institute for the Advanced Study of Human Biology (WPI-ASHBi), Kyoto University, Kyoto, Japan

[8] Department of Medicine, Indiana University, Indianapolis, Indiana, USA

Running Title: Megalin, Atherosclerosis, and TIN

*These authors contributed equally

Corresponding Authors:
Alan Daugherty
741 S. Limestone
BBSRB Room B243
Lexington, KY
Telephone: +1-859-323-3512
E-mail: Alan.Daugherty@uky.edu

Hong S. Lu
741 S. Limestone
BBSRB Room B249
Lexington, KY
Telephone: +1-859-323-4639
E-mail: Hong.Lu@uky.edu

MAJOR RESOURCES TABLES

Table I. Breeding strategy for *Ndrg1-Cre ERT2* × *Lrp2* f/f mice

	Male parent	Female parent	Offsprings
F0	*Ndrg1-Cre ERT2* +/0	*Lrp2* f/f	Female f/+ x *Ndrg1-Cre ERT2* 0/0<br>Male f/+ x *Ndrg1-Cre ERT2* +/0
F1	*Ndrg1-Cre ERT2* +/0<br>*Lrp2* f/+	*Ndrg1-Cre ERT2* 0/0<br>*Lrp2* f/+	Female f/f x *Ndrg1-Cre ERT2* 0/0<br>Male f/f x *Ndrg1-Cre ERT2* +/0
F2	*Ndrg1-Cre ERT2* +/0<br>*Lrp2* f/f	*Ndrg1-Cre ERT2* +/0<br>*Lrp2* f/f	f/f x *Ndrg1-Cre ERT2* 0/0<br>f/f x *Ndrg1-Cre ERT2* +/0<br>Both males and females-Experiments

Table II. Animals (in vivo studies) – In House Breeding (Littermates)

Mouse Strain	Background Strain	Sex
Ndrg1-Cre ERT2 0/0 *Lrp2* f/f (PTC-LRP2 +/+)	LDL Receptor -/-	Male & Female
Ndrg1-Cre ERT2 +/0 *Lrp2* f/f (PTC-LRP2 -/-)	LDL Receptor -/-	Male & Female
Ndrg1-Cre ERT2 0/0 *Lrp2* f/f (PTC-LRP2 +/+)	C57BL/6J	Male & Female
Ndrg1-Cre ERT2 +/0 *Lrp2* f/f (PTC-LRP2 -/-)	C57BL/6J	Male & Female

Table III. Diets from Inotiv used in mouse studies

Diet #	2918 (Normal Laboratory Diet)	TD.88137 (Western Diet)
Carbohydrate (kcal/kcal%)	58	42.7
Protein (kcal/kcal%)	24	15.2
Fat (kcal/kcal%)	18	42

Table IV. Primary Antibodies for Immunostaining

Antibody	Vendor	Cat #	Working Concentration
Rabbit anti-mouse/rat angiotensinogen (AGT)	IBL-America	28101	1 µg/ml
Rabbit anti-CD68 (E307V)	Cell Signaling Technology	97778	0.1 µg/mL
Rabbit anti-megalin	Abcam	ab76969	0.5 µg/ml

Secondary Antibodies for Immunostaining

Antibody	Vendor	Cat #
ImmPRESS goat anti-rabbit IgG	Vector	MP-7451

ARRIVE Essential 10 Checklist

Item	Application
Ethics	Approved by the University of Kentucky IACUC (2018-2968).
Sex	Described in each figure.
Inclusion criteria	Based on sex, age, body weight, and overt health appearance in each experiment.
Exclusion criteria	Based on sex, age, body weight, or medical cases reported by a veterinarian.
Sample size	Described in each figure legend.
Sample size calculation	None
Primary endpoint	Atherosclerosis or renal pathologies
Randomization	Study mice were numbered and grouped randomly
Blinding	None for experimental design. Some data were measured by two investigators independently to validate the consistency of measurements.
Statistical analysis	SigmaPlot 15.0 (SYSTAT Software Inc., CA) or R Statistical Software.
Statistical method	Described in each figure legend
Data availability	All numerical data used for figures are available in the Supplemental Excel File.

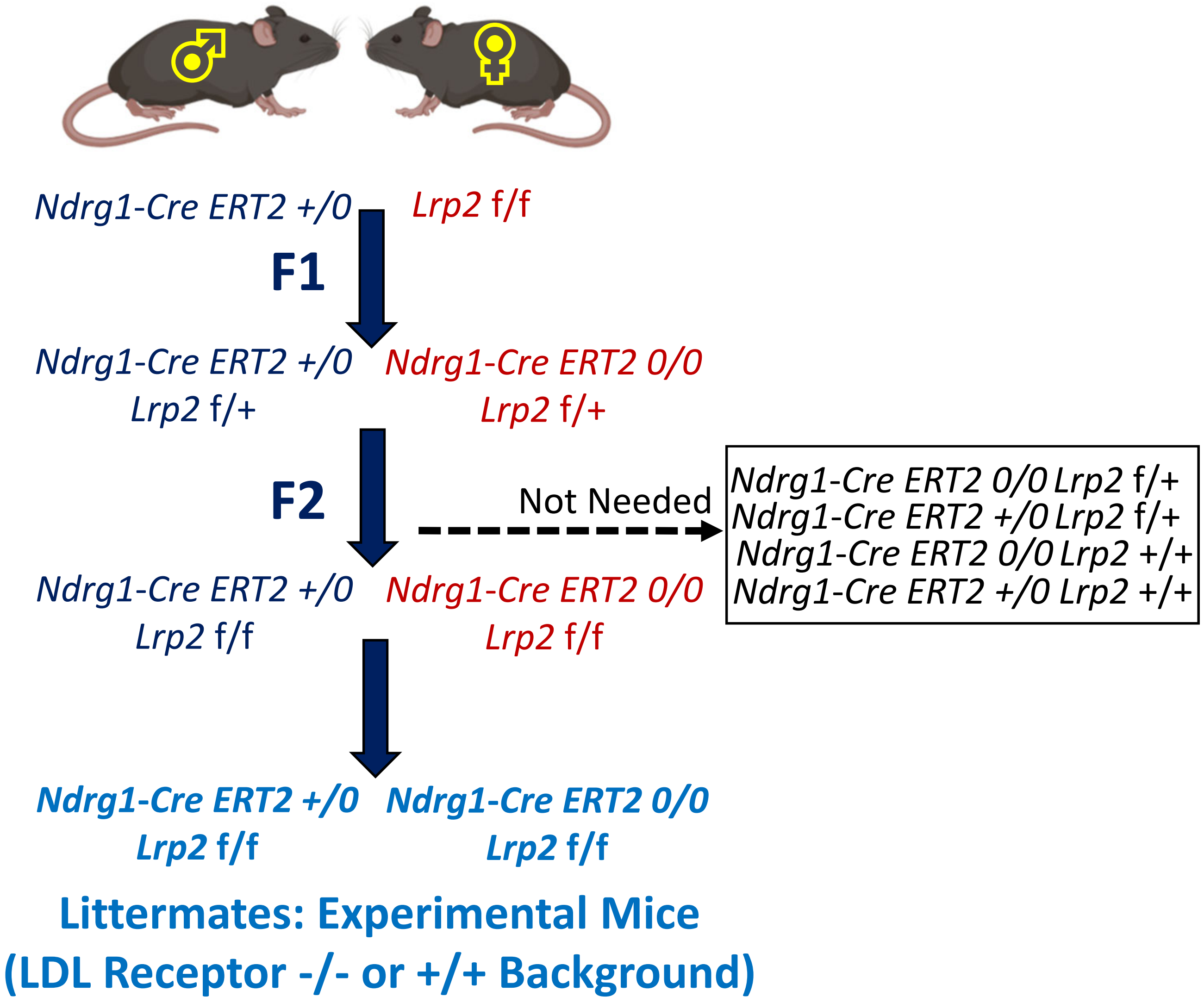

Figure S1. Breeding strategy to develop inducible PTC-LRP2 +/+ and PTC-LRP2 -/- littermates for experiments. Study mice were either LDL receptor +/+ or -/- on a C57BL/6J background as indicated in each figure. PTC-specific deletion of megalin was induced by intraperitoneal injection of tamoxifen for 5 consecutive days when they were 4-6 weeks old.

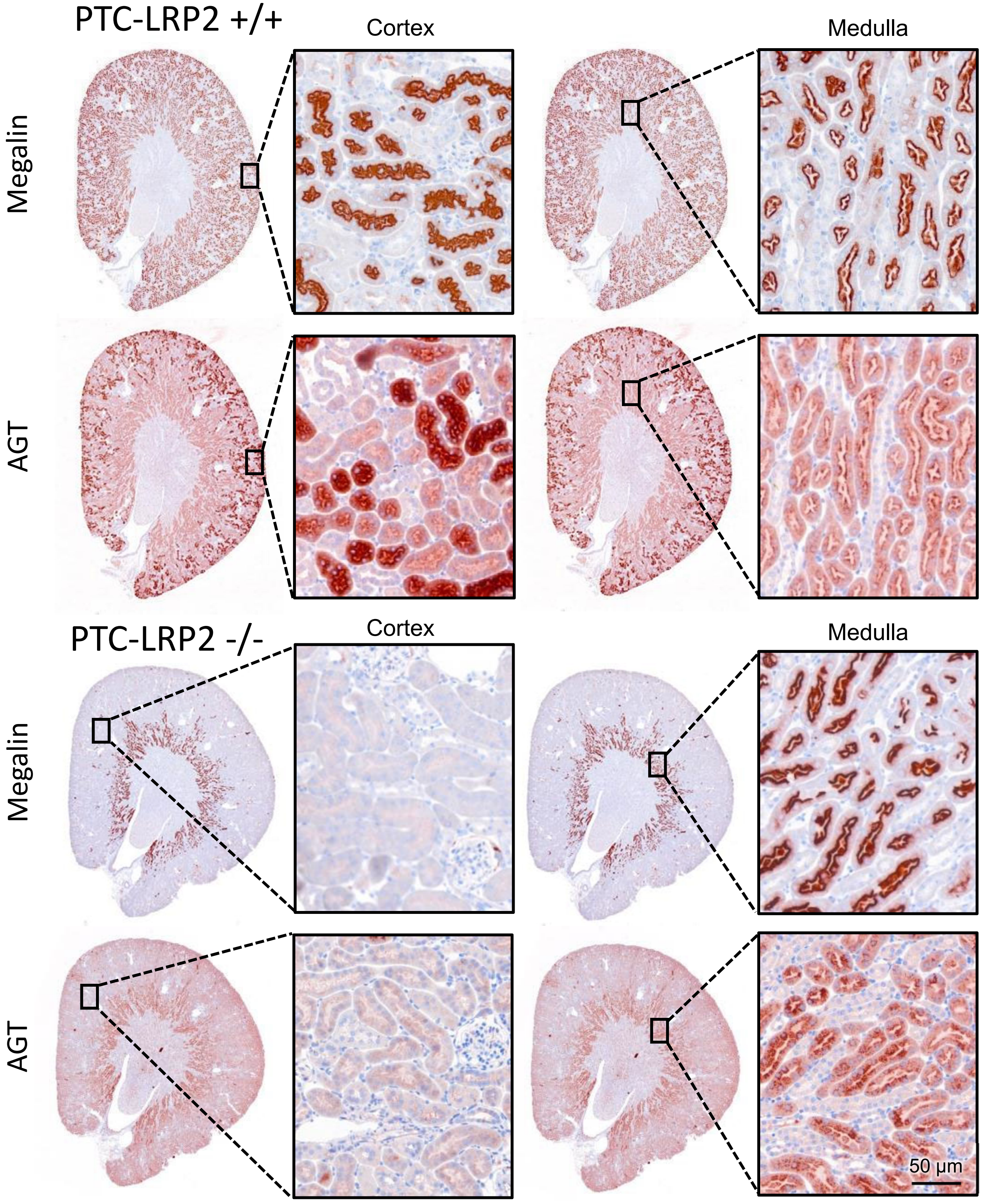

Figure S2. Immunostaining of megalin and AGT in PTC-LRP2 +/+ and PTC-LRP2 -/- mice. Immunostaining of megalin or AGT. Four to six-week-old male mice in an LDL receptor -/- background received intraperitoneal injections of tamoxifen for 5 consecutive days. Kidney tissues were harvested and sectioned at 14 weeks after completing the tamoxifen injection.

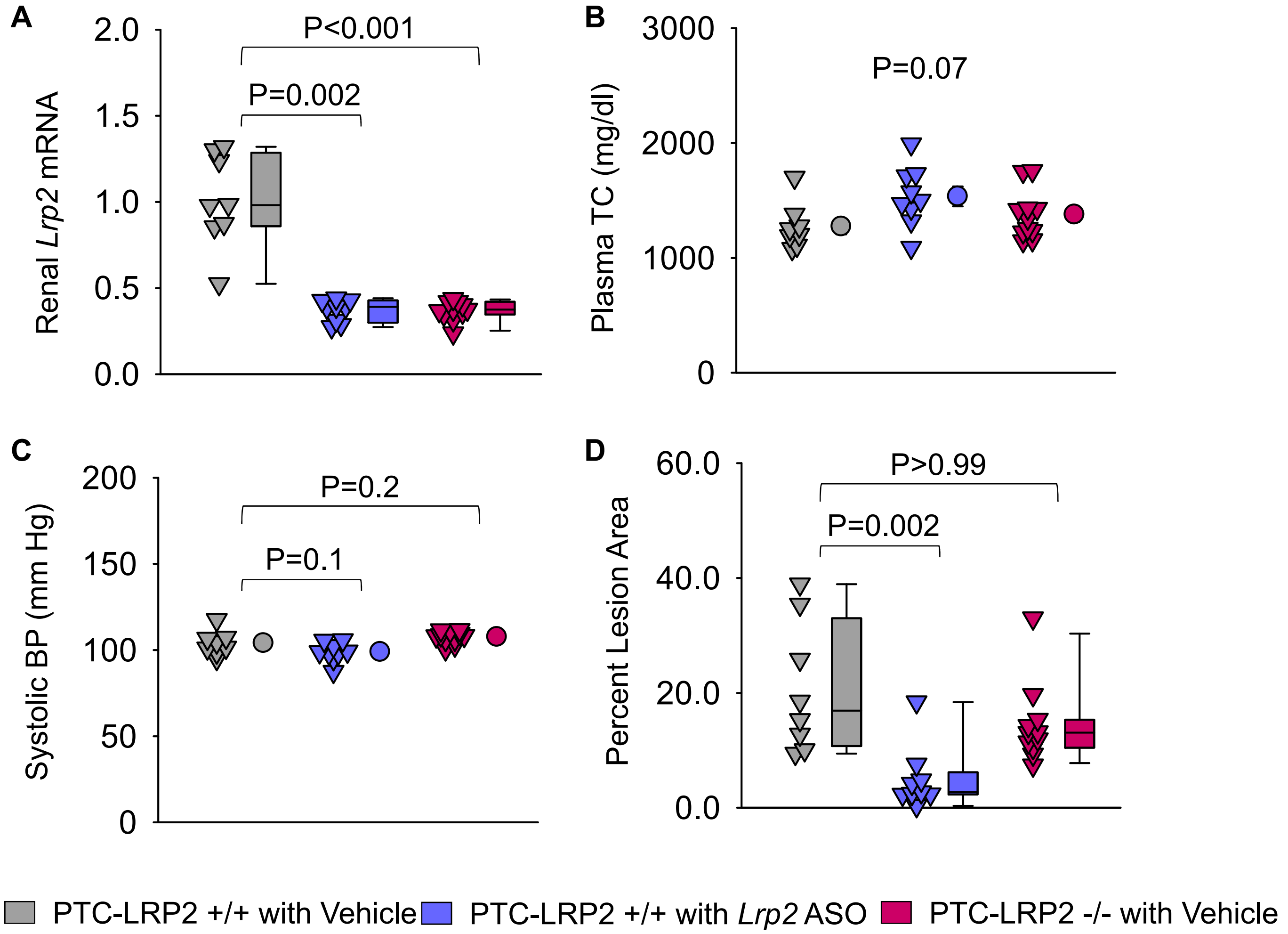

Figure S3. Renal PTC-specific megalin deletion did not attenuate atherosclerosis in female mice. Female PTC-LRP2 +/+ and PTC-LRP2 -/- mice on an LDL receptor -/- background were fed a Western diet for 12 weeks. **(A)** Renal *Lrp2* mRNA was quantified by qPCR and normalized by the geometric mean of 3 reference genes: *Actb*, *Gapdh*, and *Rplp2*. **(B)** Plasma TC (total cholesterol) concentrations were measured using an enzymatic method. **(C)** Systolic BP (blood pressure) was measured using a tail-cuff system. **(D)** Atherosclerosis was measured and quantified using an *en face* method. Statistical analyses: Kruskal-Wallis one-way ANOVA on ranks followed by the Dunn method (**A** and **D**) or one-way ANOVA followed by the Holm-Sidak test (**B** and **C**).

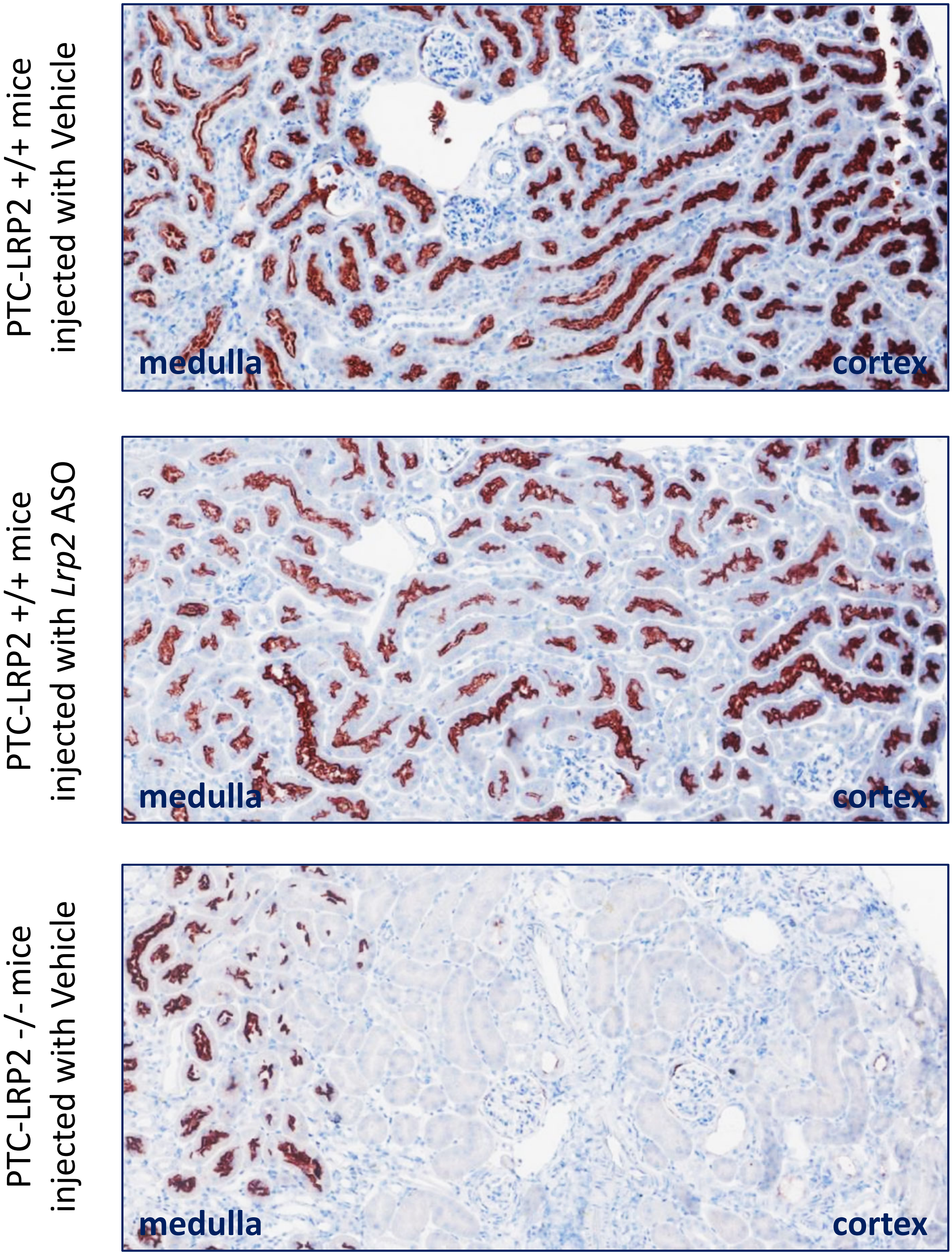

Figure S4. Immunostaining of megalin. Renal PTC-specific megalin deletion did not attenuate atherosclerosis in female mice. Male PTC-LRP2 +/+ injected with PBS (vehicle) or *Lrp2* ASO and PTC-LRP2 -/- mice injected with vehicle.

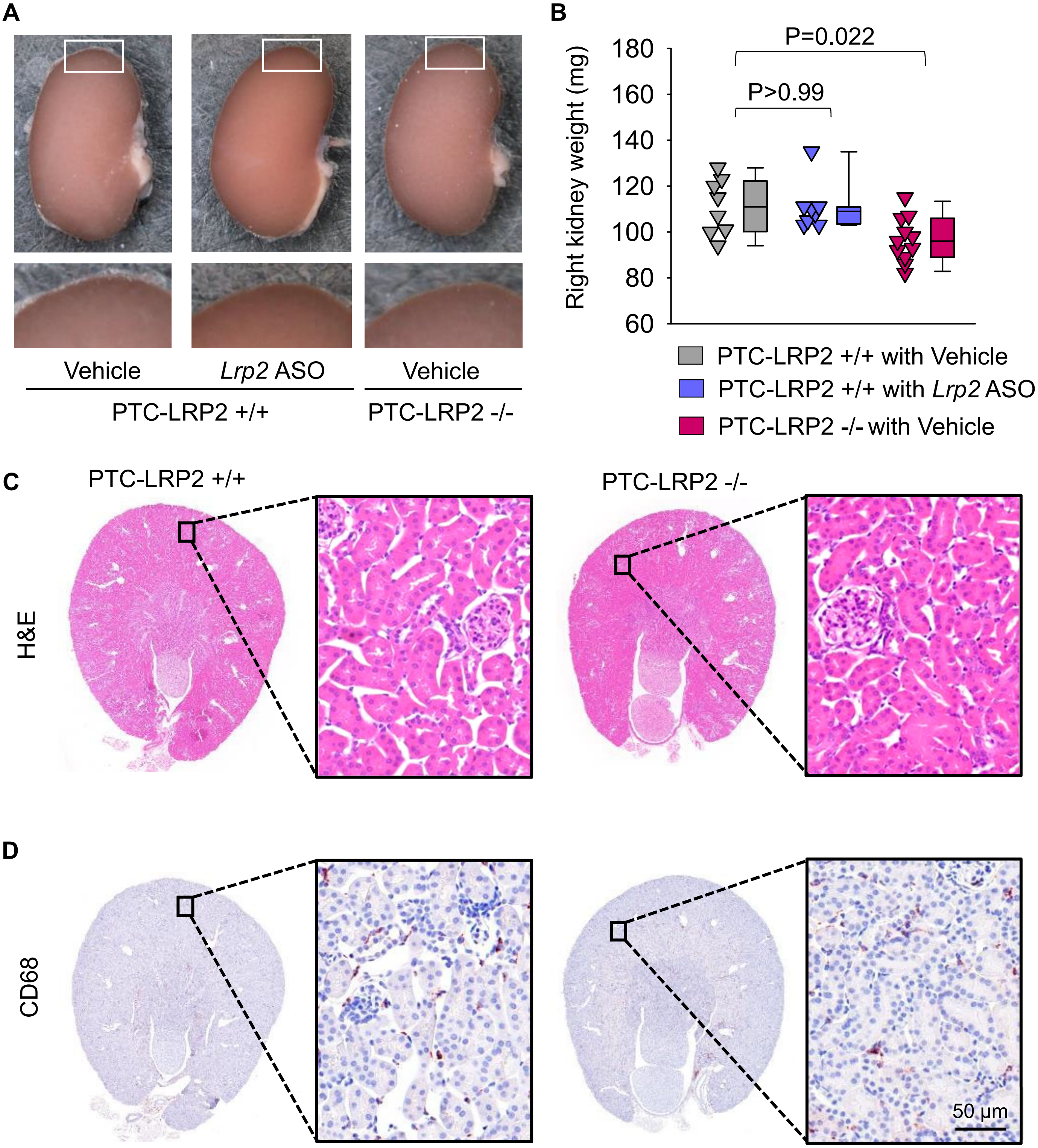

Figure S5 Renal PTC-specific megalin deletion did not cause significant pathology in female LDL receptor -/- mice. Female PTC-LRP2 +/+ and PTC-LRP2 -/- mice in an LDL receptor -/- background were fed a Western diet for 12 weeks. Gross images of kidneys **(A)** and kidney weight **(B)** at termination. **(D)** H&E staining and **(E)** immunostaining of megalin in cross-sections of mouse kidney. Statistical analysis: Kruskal–Wallis one-way ANOVA on Ranks test followed by Dunn's test (**B**).

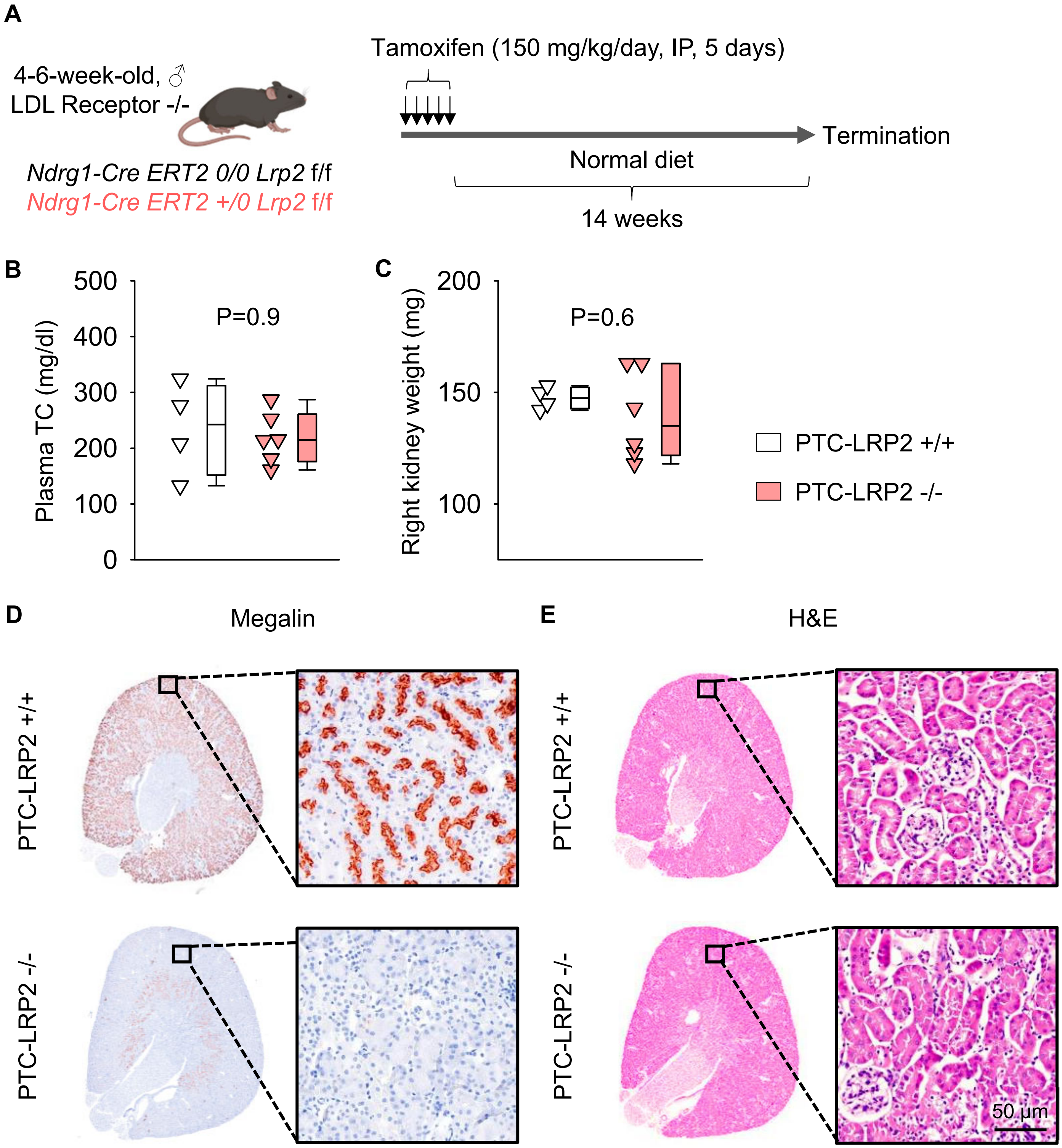

Figure S6. Renal PTC-specific megalin deletion did not cause significant renal pathology in male LDL receptor -/- mice fed a normal diet. (A) Male PTC-LRP2 +/+ and PTC-LRP2 -/- mice in an LDL receptor -/- background were fed a normal laboratory rodent diet for 15 weeks after tamoxifen induction. **(B)** Plasma TC (total cholesterol) concentrations were measured using an enzymatic method. **(C)** Kidney weight was measured at termination. **(D)** Immunostaining of megalin in cross-sections of mouse kidneys. **(E)** H&E staining in cross-sections of mouse kidney. Statistical analysis: Mann-Whitney U-test (**B** and **C**).

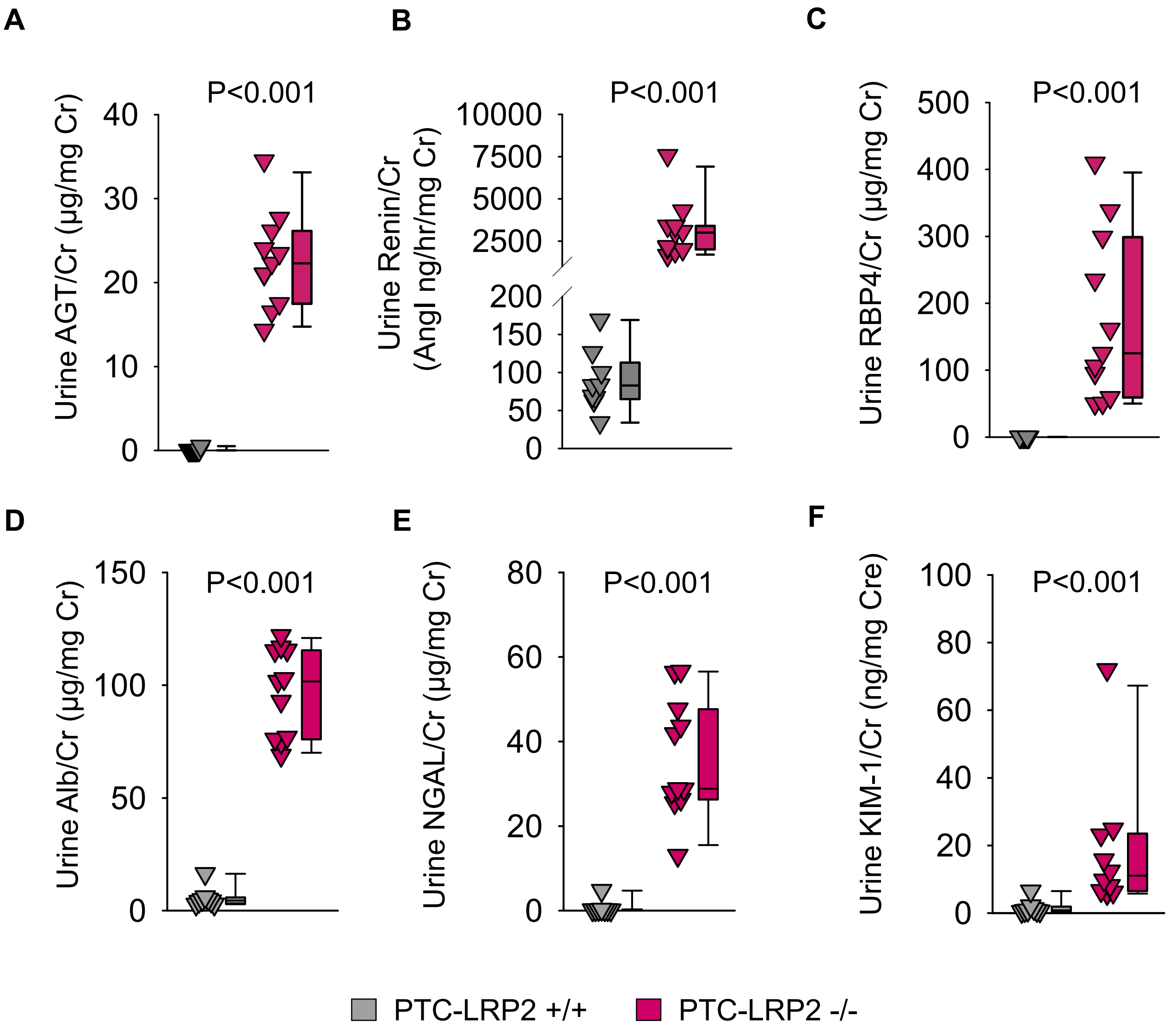

Figure S7. Renal PTC-specific megalin deletion led to increased renal injury markers in female mice. Female PTC-LRP2 +/+ and PTC-LRP2 -/- mice in an LDL receptor -/- background were fed a Western diet for 12 weeks. AGT **(A)**, renin **(B)**, RBP4 **(C)**, albumin **(D)**, NGAL **(E)**, and KIM-1 **(F)** in urine were measured using ELISA kits and normalized by urine creatinine (Cr) concentrations. Statistical analysis: Mann-Whitney U test.

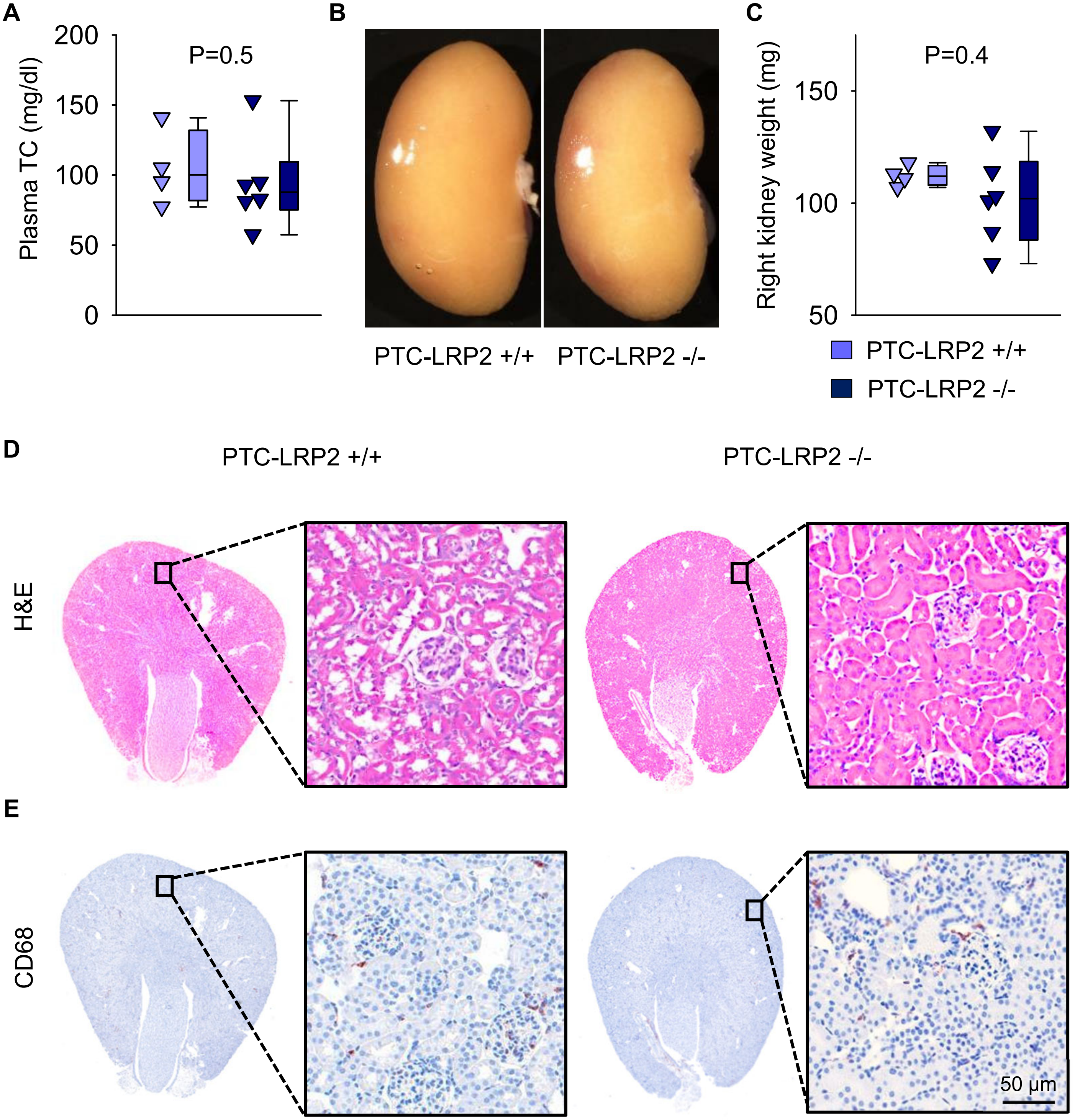

Figure S8. Renal PTC-specific megalin deletion did not cause significant pathological changes in female LDL receptor +/+ mice. Female PTC-LRP2 +/+ and PTC-LRP2 -/- mice on a C57BL/6J background were fed a Western diet for 12 weeks. **(A)** Plasma TC (total cholesterol) concentrations were measured using an enzymatic method. Gross images of kidneys **(B)** and kidney weight **(C)** at termination. **(D)** H&E staining and **(E)** immunostaining of CD68 in tissue sections of mouse kidneys. Statistical analysis: Mann-Whitney U-test (**A** and **C**).

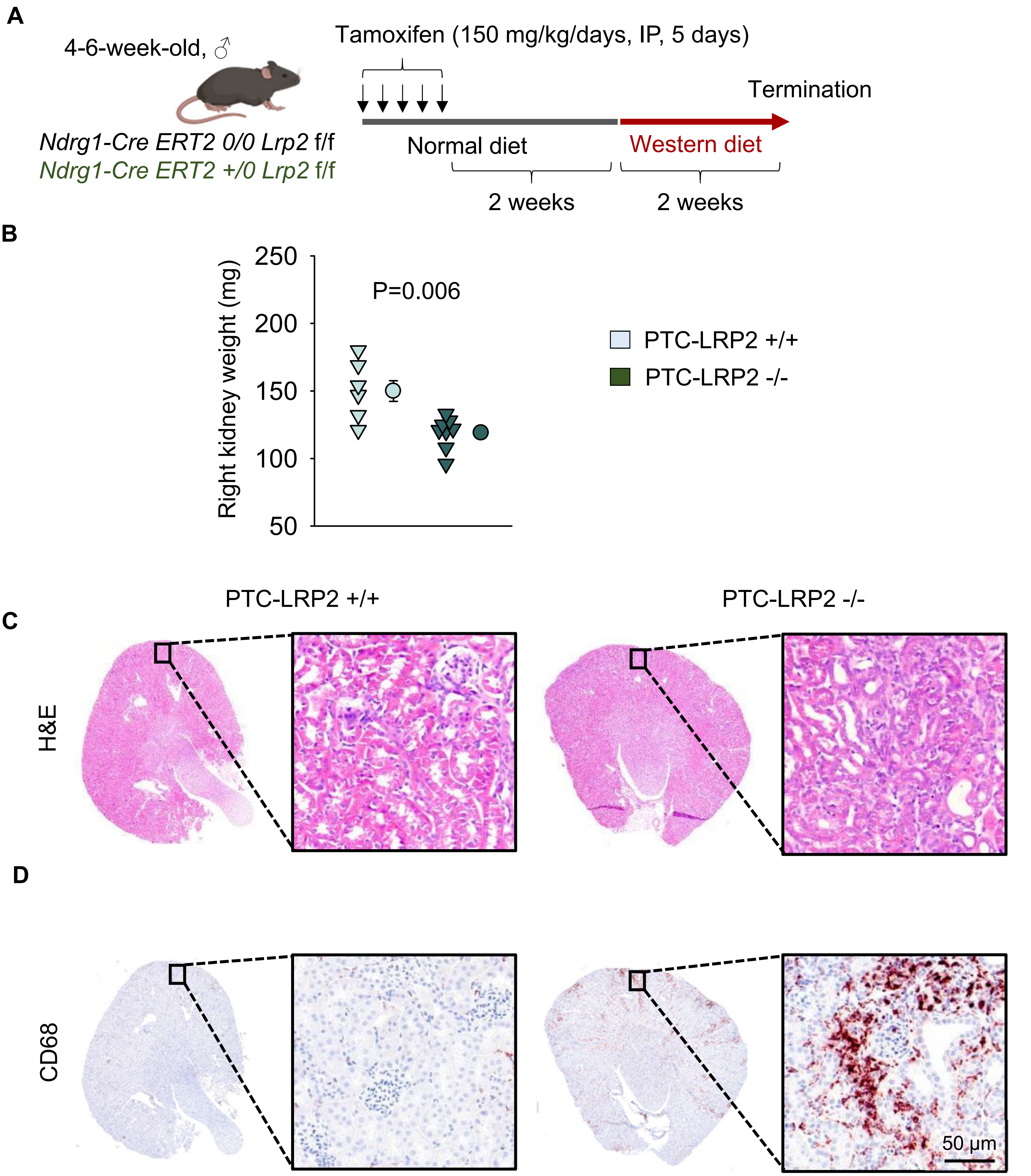

Figure S9. Renal PTC-specific megalin deletion caused renal pathologies in male LDL receptor +/+ mice. Male PTC-LRP2 +/+ and PTC-LRP2 -/- mice on a C57BL/6J background were fed a Western diet for 2 weeks **(A)**. **(B)** Kidney weight was measured at termination and analyzed by Student's t-test. **(C)** H& E staining and **(D)** immunostaining of CD68 in cross-sections of mouse kidneys.
